# Resveratrol Stabilization and Loss by Sodium Caseinate, Whey and Soy Protein Isolates: Loading, Antioxidant Activity, Oxidability

**DOI:** 10.3390/antiox11040647

**Published:** 2022-03-28

**Authors:** Xin Yin, Hao Cheng, Wusigale, Huanhuan Dong, Weining Huang, Li Liang

**Affiliations:** 1 State Key Lab of Food Science and Technology, Jiangnan University, Wuxi 214122, China; 7190112057@stu.jiangnan.edu.cn (X.Y.); haocheng@jiangnan.edu.cn (H.C.); 17865917062m@sina.cn (H.D.); wnhuang@jiangnan.edu.cn (W.H.); 2 School of Food Science and Technology, Jiangnan University, Wuxi 214122, China; 3 Key Laboratory of Dairy Biotechnology and Engineering, Ministry of Education, Inner Mongolia Agricultural University, Hohhot 010018, China; wusigale@imau.edu.cn; 4 Inner Mongolia Key Laboratory of Dairy Biotechnology and Engineering, Hohhot 010018, China

**Keywords:** protein, resveratrol, loading, antioxidant activity, oxidability, stability

## Abstract

The interaction of protein carrier and polyphenol is variable due to their environmental sensitivity. In this study, the interaction between resveratrol and whey protein isolate (WPI), sodium caseinate (SC) and soy protein isolate (SPI) during storage were systematically investigated from the aspects of polyphenol loading, antioxidant activity and oxidability. It was revealed that resveratrol loaded more in the SPI core and existed both in the core of SC micelles and on the particle surface, while WPI and resveratrol mainly formed in complexes. The loading capacity of the three proteins ranked in order SC > SPI > WPI. ABTS assay showed that the antioxidant activity of the protein carriers in the initial state was SC > SPI > WPI. The results of sulfhydryl, carbonyl and amino acid analysis showed that protein oxidability was SPI > SC > WPI. WPI, with the least oxidation, improved the storage stability of resveratrol, and the impact of SC on resveratrol stability changed from a protective to a pro-degradation effect. Co-oxidation occurred between SPI and resveratrol during storage, which refers to covalent interactions. The data gathered here suggested that the transition between the antioxidant and pro-oxidative properties of the carrier is the primary factor to investigate its protective effect on the delivered polyphenol.

## 1. Introduction

Protein-based assemblies including molecular complexes, nano-/micro-particles, and their stabilized emulsions and emulsion gels have been expected to protect antioxidants [1,2]. Even though the stabilization mechanism of polyphenols in proteins is not fully clear, there is a hypothesis that proteins express a protective effect by shielding the environmental accessibility of polyphenols and scavenging the free radical [3]. However, amphiphilic and hydrophilic polyphenols cannot be completely encapsulated in a carrier, and a portion of polyphenols are still in the free form. Meanwhile, it is worth noting that antioxidants can be converted into pro-oxidants under certain conditions and proteins may generate reactive oxidative species [4]. The imbalance between pro-oxidation and anti-oxidation in the physiological system eventually leads to the oxidation of biomolecules, the so-called oxidative stress [5]. The interaction between proteins and polyphenols might also be complicated and changeable, since they are both environment sensitive.

It has been reported that bioactive components including vitamins, polyunsaturated fatty acids and polyphenols may also affect the delivery carriers. The photo-decomposition of folic acid caused the indirect oxidation of the whey protein isolate (WPI), which enhanced the protein antioxidant activity, leading to increased protection for the folic acid [6]. β-Carotene, one of the major carotenoids, produced free radicals that can accelerate the oxidation of WPI in oil-in-water emulsions [7], while phenolic anthocyanins provided protection against the oxidation of Trp [8]. The oxidation of fish protein rich in polyunsaturated fatty acids was promoted by lipid oxidation products, especially secondary oxidation products, and the oxidation of protein and lipids occurred in parallel, showing a good correlation [9]. Although protein–polyphenol interaction has been investigated for the modification of protein structure and colloidal stability [10,11], its impact on the stability of polyphenols has rarely been reported until now. It is thus necessary to clarify the mechanism of proteins on the stability of polyphenols in more depth.

Resveratrol (*trans*-3,5,4′-trihydroxy-stilbene) is known as a polyphenolic compound with antioxidant activity. However, resveratrol is prone to oxidation, which limits its application in commercial products. Various proteins (e.g., zein, gliadin and ovalbumin) have been reported to stabilize resveratrol [12,13], but bovine serum albumin (BSA) accelerates the degradation of resveratrol [14]. Proteins in the molecular level and in the form of micelles might provide a different microenvironment and unique carrying properties for targeted antioxidants [15]. β-casein in the molecular level improved the storage stability of both *cis-* and *trans*-resveratrol better than β-casein micelles, although β-casein micelles could inhibit the transformation of resveratrol from *trans*-isomer to *cis*-isomer to a certain extent [16]. The stabilization effect on resveratrol is dependent on the type and concentration of protein carriers [17], but lacking systematic comparison study. Therefore, there is a growing demand for clarifying the theoretical basis to select suitable proteins as carrier materials for resveratrol.

Sodium caseinate (SC) and WPI are major milk proteins, and SC has a disordered structure and is more hydrophobic properties than WPI, while WPI contains two major globular proteins β-lactoglobulin and α-lactalbumin [18]. Soy protein isolate (SPI) is mainly composed of 7S and 11S globulins. WPI, SC and SPI are generally recognized as safe (GRAS), and their assemblies are commonly used to protect antioxidants against oxidation and degradation [19,20]. In the present study, WPI, SC, and SPI at various concentrations were used to investigate their effect on the storage stability of resveratrol and the polyphenol impact on the composition of proteins. The data gathered here should help guide the shelf life of the protein–polyphenol system used in commercial products.

## 2. Materials and Methods

### 2.1. Materials

WPI (≥92%) was obtained from Davisco International Inc (Le Sueur, MN, USA). SPI (≥90%) was from Shandong Xiya Chemical Industry Co., Ltd. (Linshu, Shandong, China). SC, resveratrol (trans-isomer, >99%) and polydatin (HPLC grade, >95%) were purchased from Sigma-Aldrich Co. (St. Louis, MO, USA). 2,2′-azino-bis-3- ethylbenzthiazoline-6-sulphonic acid (ABTS) was purchased from Aladdin Bio-Chem Technology Co., Ltd. (Shanghai, China). Other agents were of analytical grade and purchased from SinoPharm CNCM Ltd. (Shanghai, China).

### 2.2. Sample Preparation

WPI, SPI or SC powder was dissolved in ultrapure water. The solutions were adjusted to pH 12 with 2 M NaOH and hydrated fully with magnetic stirring for 1 h, and then neutralized the pH to 7 with 2 M HCl under agitation for another 1 h. Stock solutions of proteins were 0.02%, 0.2% and 2.0% (*w*/*v*). Stock solution of resveratrol was prepared at a concentration of 2 mM by dissolving in 70% (*v*/*v*) ethanol. The resveratrol solution was added into protein solutions and diluted with water at pH 7 under stirring for 30 min. The final concentrations were 0.01%, 0.10% and 1.00% for proteins and 25, 50 and 100 μM for resveratrol. The 0.02% (*w*/*v*) sodium azide was added to solutions as an antimicrobial agent.

### 2.3. Fluorescence Spectroscopy

Fluorescence of pyrene as a probe was measured on a Cary Eclipse fluorescence spectrophotometer (Agilent Co., Ltd., New York, NY, USA) equipped with 10 mm quartz cuvettes. The spectral resolution was 2.5 nm for both excitation and emission. Pyrene in acetone was added into samples with its final concentration of 1 μM under stirring for at least 12 h before measurement. Fluorescence emission spectra were scanned from 350 to 600 nm with the excitation wavelength of 335 nm and the ratio of the intensity of the first and third bands (I_1_/I_3_) was calculated [21].

Fluorescence emission spectra of resveratrol in the absence or presence of proteins were recorded from 330 to 600 nm at an excitation wavelength of 320 nm. Slit widths with a nominal band-pass of 5 nm were used for both excitation and emission. Background of proteins was subtracted from raw spectra.

### 2.4. Particle Size and ζ-Potential

Size distribution by the intensity and ζ-potential were determined by a NanoBrooker Omni Particle size Analyzer (Brookhaven Instruments Ltd., New York, NY, USA) with a He/Ne laser (λ = 633 nm) at a scattering angle of 173°. They were obtained using an NNLS model and Smoluchowski model through phase analysis light-scattering (PALS) measurement, respectively.

### 2.5. Color Evaluation

The color parameters of protein-resveratrol solutions before and after storage at 45 °C for 30 days were measured using a ColorQuest XE colorimeter (ColorQuest XE, Hunter Lab, Reston, VA, USA) and calculated using the Hunter Lab color scale (L*a*b*). L* represents the lightness (black = 0 to white = 100), a* varies from red (positive) to green (negative), and b* varies from yellow (positive) to blue (negative). The total color difference (ΔE) was calculated from the tristimulus color coordinates using the following equation:

(1) $$\Delta E={\left[{\left(L^{*}-L_{i}^{*}\right)}^{2}+{\left(a^{*}-a_{i}^{*}\right)}^{2}+{\left(b^{*}-b_{i}^{*}\right)}^{2}\right]}^{1/2}$$

where, L_i_*, a_i_*, b_i_* are the initial values of the CIE L*a*b* color coordinates of freshly-prepared samples, and L*, a*, b* are the color coordinates of samples after 30 days. Additionally, the difference in chroma (ΔC*) value, which represents the color intensity of samples, was analyzed by the following equation [22]:

(2) $$\Delta C^{*}={\left[{\left(a^{*}-a_{i}^{*}\right)}^{2}+{\left(b^{*}-b_{i}^{*}\right)}^{2}\right]}^{1/2}$$

### 2.6. Resveratrol Quantification

An exactly 0.5 mL sample was mixed with 0.5 mL polydatin (internal standard, 50 μM) in methanol and then added into 4 mL methanol under vortexing for 60 s. After the mixture was centrifuged at 15,000× *g* for 60 min, the supernatant was measured on the Alliance HPLC system equipped with a 2695 separation module and 2998 PDA detector (Waters, Milford, MA, USA). The mobile phase was a mixture of methanol and distilled water (50:50, *v*/*v*), the flow rate was 1 mL min^−1^, and the column temperature was 35 °C. Both *trans*-resveratrol and polydatin were analyzed at 306 nm [23].

### 2.7. Loading Efficiency

Loading efficiency of resveratrol was determined by isoelectric precipitation method [24]. The samples with WPI, SPI and SC were adjusted to pH 4.8–4.6 using 0.1 M NaOH or HCl. Loading efficiency of resveratrol was calculated according to following formulation:

(3) $$\mathrm{Loading}\;\mathrm{efficiency}\left(\%\right)=\left(1-\frac{C_{s}}{C_{0}}\right)\times 100$$

where, C_0_ and C_s_ were resveratrol in samples and in the supernatant, centrifuged at 5000× *g* for 20 min, respectively.

### 2.8. Antioxidant Activity

ABTS assay was analyzed according to previous methods [25]. In brief, 7.4 mM ABTS and 2.6 mM K_2_S_2_O_8_ were mixed in the dark for 12 h to produce ABTS⋅^+^ solution, which was diluted and mixed with samples or buffer at a volume ratio of 19:1 and kept in the dark for 6 min. The absorbance was measured at 729 nm using a UV-1800 UV–Vis spectrophotometer (Shimadzu Co., Tokyo, Japan). The radical-scavenging activity was calculated as follows:

(4) $$\mathrm{Scavenging}\;\mathrm{capacity}\;\left(\%\right)=\frac{A_{c}-A_{s}}{A_{c}}\times 100$$

where A_c_ and A_s_ are the absorbance of radical plus buffer and sample, respectively.

### 2.9. Sulfhydryl Analysis

Samples were mixed at a volume ratio of 1:2 with 0.1 M phosphate buffer at pH 8.0 without and with 8 M urea for free and total sulfhydryl determination, respectively. Then absorbance at 412 nm was measured, after 10 mM DTNB was added under vigorous stirring and incubated in the dark for 1 h. Both reagent and sample blanks were subtracted. Content of free and total sulfhydryl was calculated by using a molar extinction coefficient of 13,600 M^−1^cm^−1^ and expressed as nmol per mg protein [26].

### 2.10. Carbonyl Analysis

Protein solutions in the presence and absence of resveratrol during storage at 45 °C were mixed at a volume ratio of 1:2 with 10 mM DNPH in 2 M HCl. After 10% (*w*/*v*) trichloroacetic acid was added and centrifuged at 10,000× *g* for 5 min, precipitate was washed with 50% ethyl acetate and then dissolved in 6 M guanidine HCl in 20 mM phosphate buffer at pH 2.3. Absorbance at 370 nm was measured and carbonyl content was calculated using an extinction coefficient of 22,000 M^−1^cm^−1^ and expressed as nmol per mg protein [27].

### 2.11. Amino Acid Analysis

Amino acids except tryptophan were analyzed through acid hydrolysis of proteins by mixing 4 mL of samples with the same volume of 12 M HCl under blown nitrogen for 3 min, followed by hydrolysis at 120 °C for 22 h. Then a certain amount of NaOH was added to neutralize, and water was added to give a total volume of 25 mL. Tryptophan was determined by alkaline hydrolysis of proteins with 10 M NaOH and neutralized with a certain amount of HCl. The supernatant was centrifuged after filtering with filter paper. Amino acids were analyzed on the Agilent 1100 HPLC system equipped with an Agilent Hypersil ODS column (Angelon Co., Ltd., New York, NY, USA). Proline was detected at 262 nm, and the other amino acids were detected at 338 nm [6].

### 2.12. Statistical Analysis

All experiments were repeated three times. Data are presented as mean ± standard deviation. An analysis of variance (ANOVA) of the data was carried out and identified using the Duncan procedure. All statistical analyses were performed using the software package SPSS 20.0 (SPSS Inc., Chicago, IL, USA). A *p* value < 0.05 was considered significant.

## 3. Results

### 3.1. Particle Characterization

**Figure 1:**
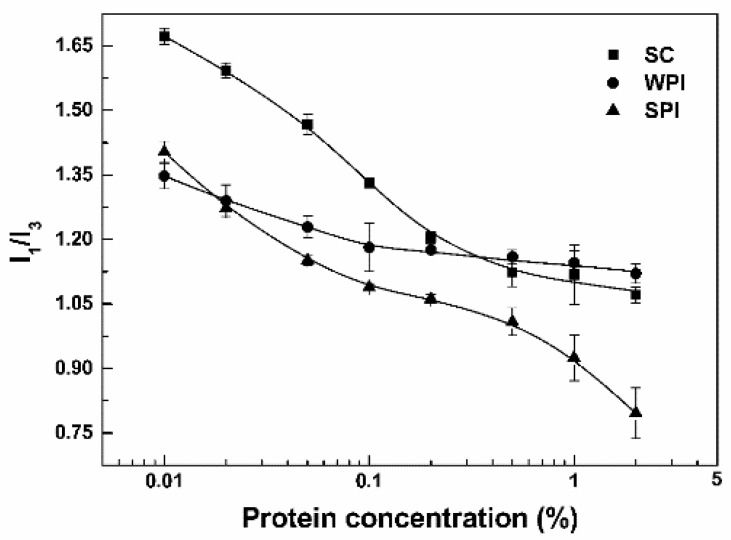
I_1_/I_3_ of whey protein isolate (WPI), sodium caseinate (SC) and soy protein isolate (SPI) solutions at various concentrations.

**Figure 2:**
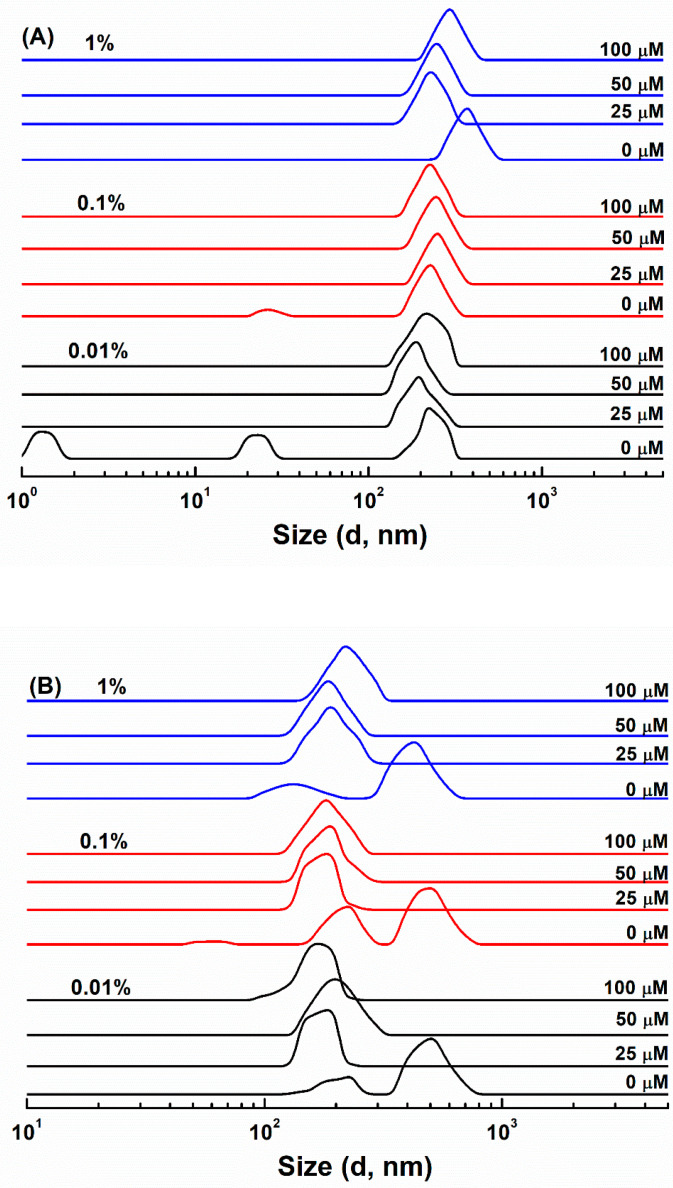

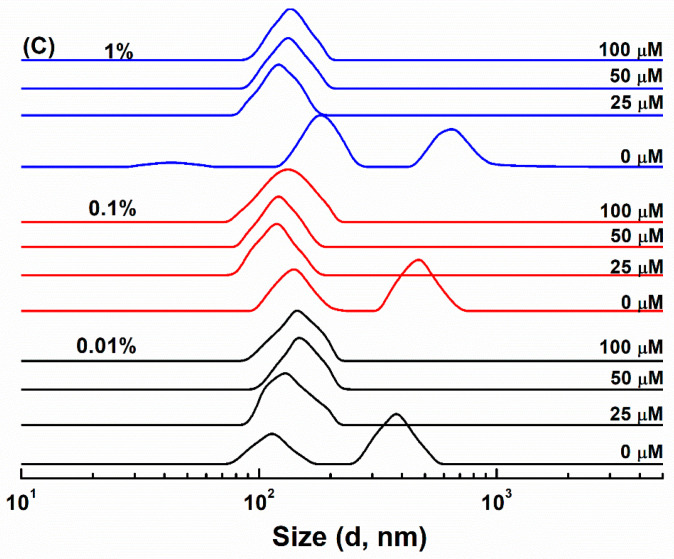
Size distribution of SC (**A**), WPI (**B**) and SPI (**C**) particles in the absence and presence of 25, 50 and 100 μM resveratrol. The protein concentrations were 0.01%, 0.1% and 1%.

Pyrene is often used to investigate the association of macromolecules and the critical micelle concentration (CMC). Its intensity ratio I_1_/I_3_ decreased as the hydrophobicity of surrounding microenvironment increased [28]. The I_1_/I_3_ ratio of pyrene in water was 1.75 (±0.01). When the concentration of SC was 0.01%, the I_1_/I_3_ ratio was 1.67 (Figure 1), and its size distribution had three peaks around 1.5, 25 and 215 nm by intensity (Figure 2A). According to the submicelle model, each casein forms small submicelle units through hydrophobic interactions, and these subunits use calcium phosphate as the cement and further aggregates together to form SC micelles [29]. The relatively low concentration of SC solution is not sufficient to drive the formation of micelles, 0.01% SC mainly dissolved in the molecular level [30]. As the protein concentrations increased above 0.5%, the I_1_/I_3_ of SC gradually decreased to about 1.10 (Figure 1). The size distribution of SC at 0.1% showed a major peak around 230 nm and a minor peak around 25 nm, while only a peak at around 380 nm was observed at 1% (Figure 2A). These results indicate that SC aggregates to form micelles at 1% concentration [31]. As for WPI and SPI, the I_1_/I_3_ ratios of 0.01% protein were respectively 1.35 and 1.40 (Figure 1). Meanwhile, WPI had two peaks around 220 and 520 nm (Figure 2B), and SPI had two peaks around 110 and 380 nm (Figure 2C). The relatively low I_1_/I_3_ and large particle size suggest that WPI and SPI had already aggregated at 0.01%. The size peaks of WPI were not dependent on its concentrations (Figure 2B), while SPI became bigger with increasing concentrations, with two major peaks around 180 and 660 nm at 1% (Figure 2C). This is consistent with the results of the I_1_/I_3_ of pyrene. From Figure 1, the I_1_/I_3_ ratios of WPI decreased slightly from 1.35 at 0.01% to 1.18 at 0.1%, and then remained unchanged as the protein concentration further increased. The I_1_/I_3_ ratios decreased as the concentrations of proteins increased, reaching around 0.80 for 2% SPI (Figure 1). By comparative analysis, these results indicate that SPI particles have the most hydrophobic core, which is consistent with the highest content of hydrophobic amino acids (Table 1 and Table 2 vs. Table 3). It has been reported that SPI had lower solubility compared with WPI and SC, while hydrophilic groups and/or water molecules were entrapped in the core of WPI particles [32,33].

**Figure 3:**
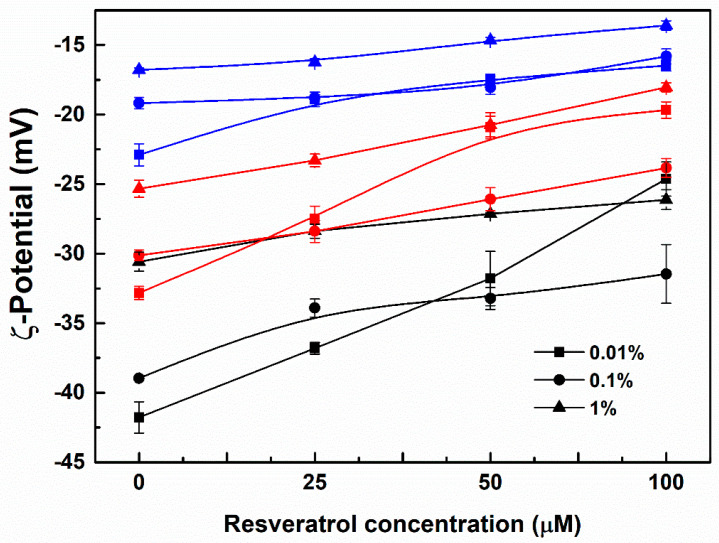
ζ−Potential of WPI (black), SC (red) and SPI (blue) particles in the absence and presence of 25, 50 and 100 μM resveratrol. The protein concentrations were 0.01%, 0.1% and 1%.

Since WPI, SC or SPI have an isoelectric point (pI) around pH 4.5~5, the ζ−potential values of their particles are negative at pH 7.0 (Figure 3). ζ-Potential absolute values of the protein particles ranked in order WPI > SC > SPI at the same concentration (Figure 3). This is consistent with their molar ratio of acidic (Asp and Glu) and basic (His, Lys and Arg) amino acids being around 2.38 for WPI, 2.03 for SC, and 1.98 for SPI, calculated from the data in Table 1, Table 2 and Table 3. Together with the most hydrophobic core of SPI particles in Figure 1, these results indicate that more negatively-charged groups were masked in SPI particles than WPI and SC particles. ζ-Potential absolute values of all complex particles decreased as the protein concentration increased (Figure 3), suggesting that negatively-charged groups were entrapped in the aggregated particle core.

ζ−Potential absolute values of the protein particles decreased as the concentration of resveratrol increased, which was most pronounced at the protein concentration of 0.01% (Figure 3). The particles of SC, WPI and SPI became more homogeneous upon loading of resveratrol (Figure 2). These results are consistent with the formation of uniform particles of WPI with naringenin, a polyhydroxy flavonoid [34]. Meanwhile, the size distribution of all protein-resveratrol particles increased as the polyphenol concentration increased (Figure 2), which is consistent with the effect of hesperetin or hesperidin concentration on their individual particles with β-conglycinin, one of the major fractions of soy proteins, possibly due to polyphenols acting as bridging agents for protein molecules [35]. SC-resveratrol, WPI-resveratrol and SPI-resveratrol particles had a size distribution around 200–300 nm, 150–250 nm and 100–200 nm, respectively. At 25 µM resveratrol, the size distribution of SC-resveratrol particles was close to the largest size distribution of SC particles (Figure 2A), while WPI-resveratrol and SPI-resveratrol particles had a size distribution close to the smallest ones of pure protein (Figure 2B,C). These results suggest that the addition of resveratrol favors the aggregation of SC but inhibits the formation of large WPI and SPI aggregates.

### 3.2. Resveratrol Loading

#### 3.2.1. Microenvironment of Resveratrol

**Figure 4:**
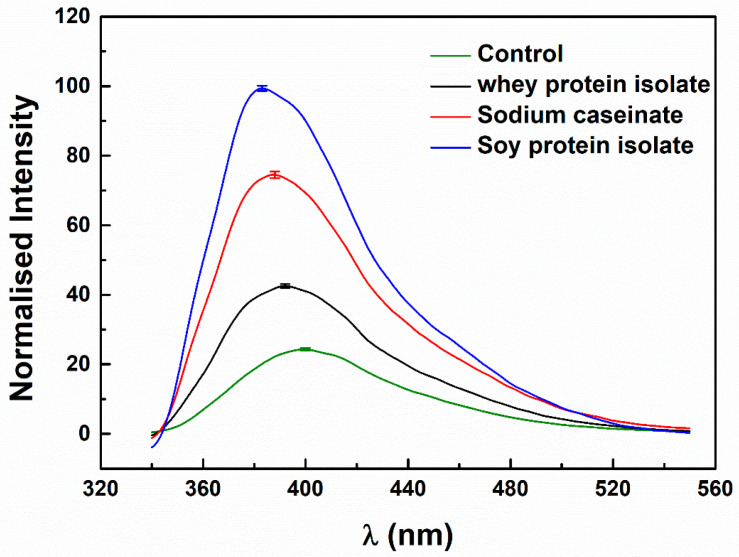
Fluorescence emission spectra of resveratrol in the absence (control) and presence of WPI, SC and SPI. Concentrations of resveratrol and proteins were 25 μM and 0.1%, respectively.

Pure resveratrol showed a crystalline structure, with sharp peaks at 6.58, 13.26, 16.39, 19.27, 22.40, 23.66, 25.28, 28.36 on a 2θ scale (Figure S2). Its characteristic peaks with less intensity were still observed in its physical mixtures with the proteins but disappeared in its protein particles (Figure S2), indicating that the polyphenol was amorphous when loaded in the protein particles [36]. From Figure 4, resveratrol in the absence of protein emits a relatively weak fluorescence, owing to its proton transfer tautomer fluorescence band [37]. The λ_max_ of resveratrol around 400 nm shifted to 392, 388 and 383 nm in the presence of 0.1% WPI, SC and SPI, respectively. At the same time, the fluorescence intensity at λ_max_ was 1.75, 3.06 and 4.09 times that of resveratrol alone. Similar changes were previously observed in the presence of β-lactoglobulin (β-LG) and bovine serum albumin (BSA), with respective fluorescence intensity at λ_max_ of 393 and 379 nm being 1.21 and 4.92 times that of resveratrol alone [38]. These results indicate that the microenvironment of resveratrol was more hydrophobic in protein particles, and the order of hydrophobicity was SPI > SC > WPI. The hydrophobicity of the resveratrol microenvironment (Figure 4) is consistent with the aggregation degree of pure protein at 1% but not that at 0.1% (SPI > WPI > SC, Figure 1). This is possibly attributed to the different impact of resveratrol loading on the aggregation of the three proteins. As discussed above, the added resveratrol as bridging agent favors the aggregation of SC (Figure 2).

#### 3.2.2. Loading Efficiency of Resveratrol

**Figure 5:**
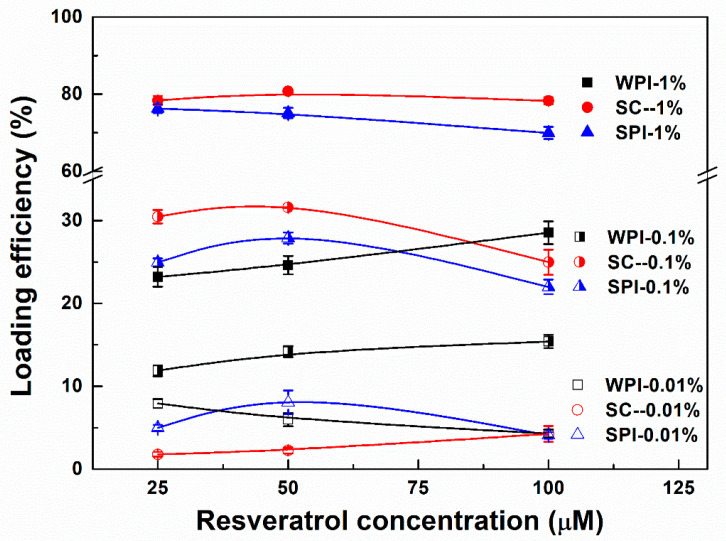
Loading efficiency of resveratrol in its complex particles with WPI (black), SC (red) and SPI (blue) at 0.01%, 0.1% and 1%.

When the concentration of proteins was 0.01%, loading efficiencies of resveratrol were between 2% and 8% (Figure 5). The polyphenol loading efficiencies at 25 and 50 µM were greater in WPI and SPI particles than in SC particles. This may be due to WPI and SPI existing in the aggregate form at 0.01%, while SC exists in the molecular state (Figure 1). The loading efficiencies of resveratrol at 100 µM were 4% in all the protein particles (Figure 5), therefore resveratrol mainly exists in the free state in the presence of 0.01% proteins. As the concentration of WPI increased from 0.1% to 1%, the loading efficiencies of resveratrol increased by around 10%, of which the highest was 28%. The highest loading efficiencies of resveratrol in the presence of 0.1% SC and SPI were 31% and 27%, which further increased at 1% SC and SPI to around 80% and 76%, respectively. In the case of 0.1% and 1% proteins, the loading efficiencies of resveratrol ranked in order SC > SPI > WPI. As mentioned above, resveratrol is conducive to the micellization of SC (Figure 2 and Figure 4). The complex of resveratrol and protein masked the charged group, and the absolute value of the ζ-potential of the system decreased in the order of WPI > SC > SPI (Figure 3). It is speculated that the loading of resveratrol in SC particles not only depends on the transfer of the hydrophobic environment, but also refers to the bridging of resveratrol to submicelles. These results supported the hypothesis that the resveratrol was mainly located in the hydrophobic core of SPI, while both entrapped in the hydrophobic core and partially bound to the surface of the SC micelles. For WPI, more resveratrol complexed with the protein. Meanwhile, the loading efficiencies of the remaining resveratrol in protein particles were similar before and after storage at 45 °C for 30 days (Figure 5 and Figure S3).

#### 3.2.3. Antioxidant Activity

**Figure 6:**
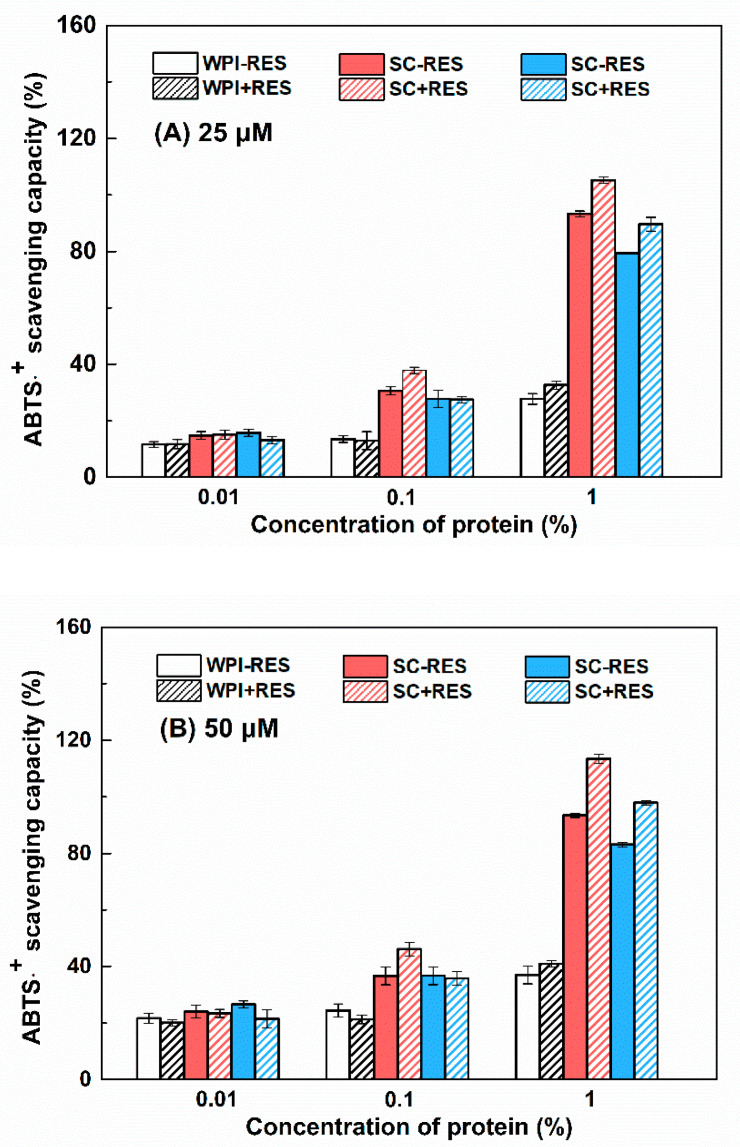

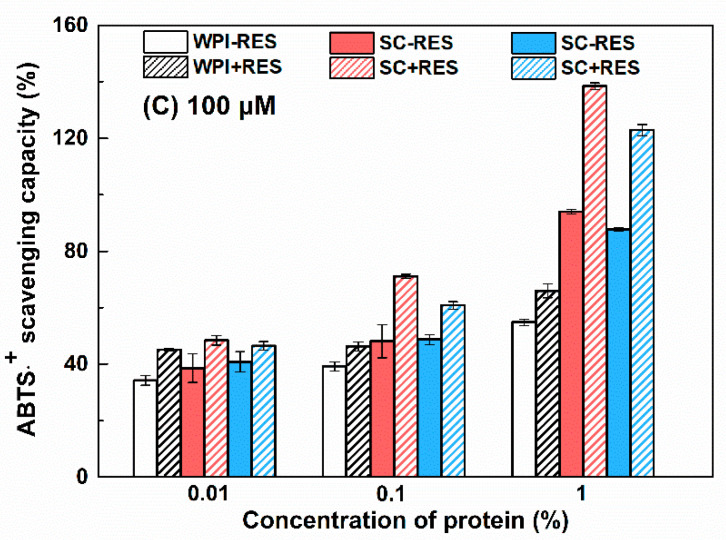
ABTS⋅^+^ scavenging capacity of resveratrol, WPI (black), SC (red), SPI (blue) and WPI-resveratrol, SC-resveratrol and SPI-resveratrol complex nanoparticles. The concentrations of proteins were 0.01%, 0.1% and 1%, while the concentrations of resveratrol were 25 (**A**), 50 (**B**) and 100 (**C**) μM.

It has been reported that casein contains more powerful antioxidant peptides than whey protein [39]. The presence of small peptides and C-terminal aromatic tyrosine residues contribute to the radical scavenging ability of SPI [40]. From Figure 6, the ABTS⋅^+^ scavenging capacity of proteins ranked in order SC > SPI > WPI under the same concentration. Resveratrol contains three phenolic hydroxyl groups and possesses antioxidant activity [41]. When the concentrations of resveratrol were 25, 50 and 100 µM, its ABTS⋅^+^ scavenging capacities were 11%, 20% and 44% (Figure 6), respectively. The scavenging capacities of WPI-resveratrol particles were similar to the sum of the individual capacity at the polyphenol concentrations of 25 and 50 µM (Figure 6A,B), suggesting an additive effect. At 100 µM, the scavenging capacities of WPI-resveratrol particles were less than the sum of the individual capacities (Figure 6C), suggesting partial screening of total antioxidant activity. As for SPI, resveratrol at 25 and 50 µM showed an additive effect with 0.01% and 0.1% protein but a masking effect with 1% protein (Figure 6A,B), and the masking effect was also observed at 100 µM resveratrol with all the investigated concentrations (Figure 6C). A masking effect was also observed in the case of SC, except for 25 and 50 μM resveratrol and 0.01% protein, which showed an additive effect (Figure 6). The masking effect is due to the protein–polyphenol interaction and the encapsulation of polyphenol in particles masking the phenolic hydroxyl groups [42,43]. It is worth noting that at 1% SPI and SC systems, the masked antioxidant activity was almost equal to that of resveratrol alone. This further confirms that resveratrol is mainly embedded in the hydrophobic core of SPI aggregations and SC micelles.

### 3.3. Protein Oxidation

#### 3.3.1. Sulfhydryl Groups

**Figure 7:**
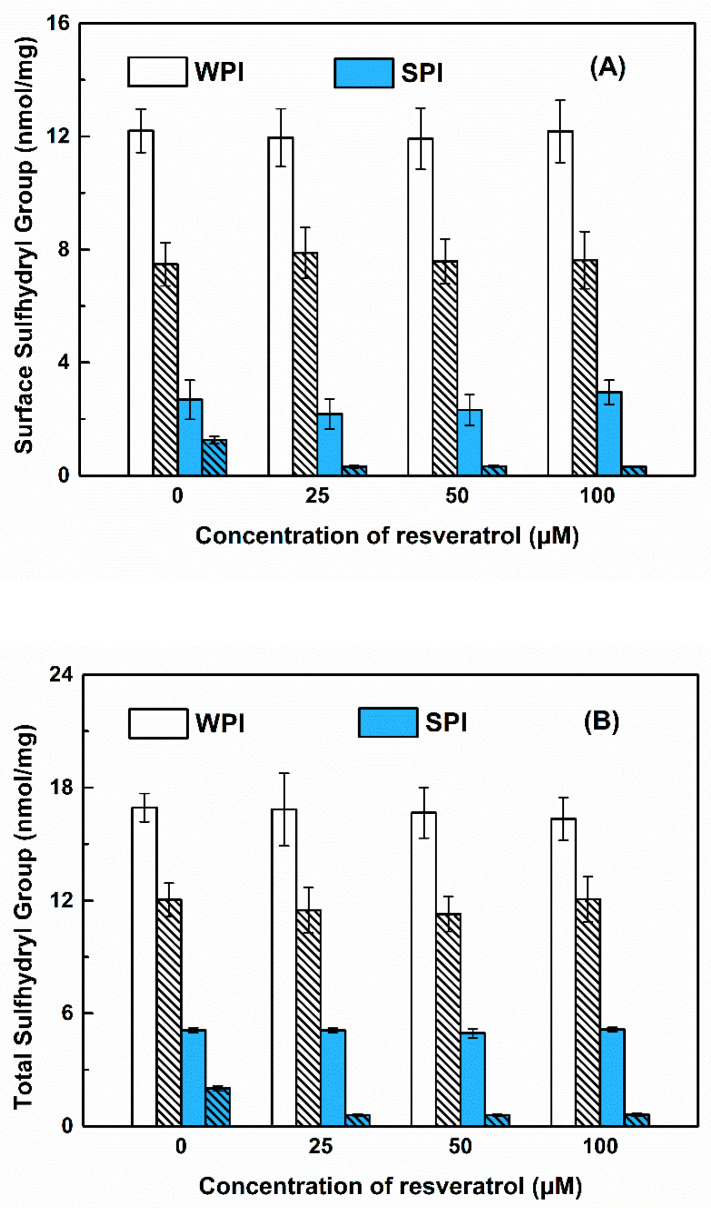
Surface (**A**) and total (**B**) sulfhydryl content of WPI-resveratrol and SPI-resveratrol complex particles before (no pattern) and after (sparse pattern) storage at 45 °C for 30 days. The concentration of proteins was 1%.

It is well known that the ability of susceptible proteins for scavenging and generating reactive oxygen radical changes with the environment, which is related to the oxidation of the protein [44]. Protein oxidation is commonly accompanied by a decrease in the number of sulfhydryl (SH) groups [45]. The surface and total sulfhydryl contents of WPI were 12 and 17 nmol/mg, respectively, and for SPI, they were 3 nmol/mg and 5 nmol/mg, respectively. The lower sulfhydryl content of SPI in the initial state compared to WPI reflects that the initial oxidation state of SPI is greater than WPI, which may be related to the protein extraction process. As reported in the process of preparing SPI from defatted soy flour, lipoxygenase (LOX) was inevitably present in the system. The weakly alkaline extraction conditions caused LOX in the soybean flour to catalyze the oxidation of residual lipids [27]. The free and total sulfhydryl content of WPI and SPI decreased after storage (Figure 7), indicating that the accessible cysteine residues located at both surface and buried in the protein were attacked by free radicals [46]. The decrease in free and total sulfhydryl contents of SPI was greater in the presence than in the absence of resveratrol, while their contents of WPI were not affected by resveratrol (Figure 7). The interference of environmental factors on WPI and SPI sulfhydryl groups has been studied. After ultrasonic treatment, the disulfide bonds of SPI were destroyed, which significantly increased the free sulfhydryl content [47]. However, sonication did not change the thiol content of the whey protein concentrate. As reported, the oxidative susceptibility of free SH groups may depend on the constituent of mixture proteins. The intramolecular positions of the free thiol groups in β-lactoglobulin and α-lactalbumin may make WPI less sensitive [48].

#### 3.3.2. Carbonyl Groups

**Figure 8:**
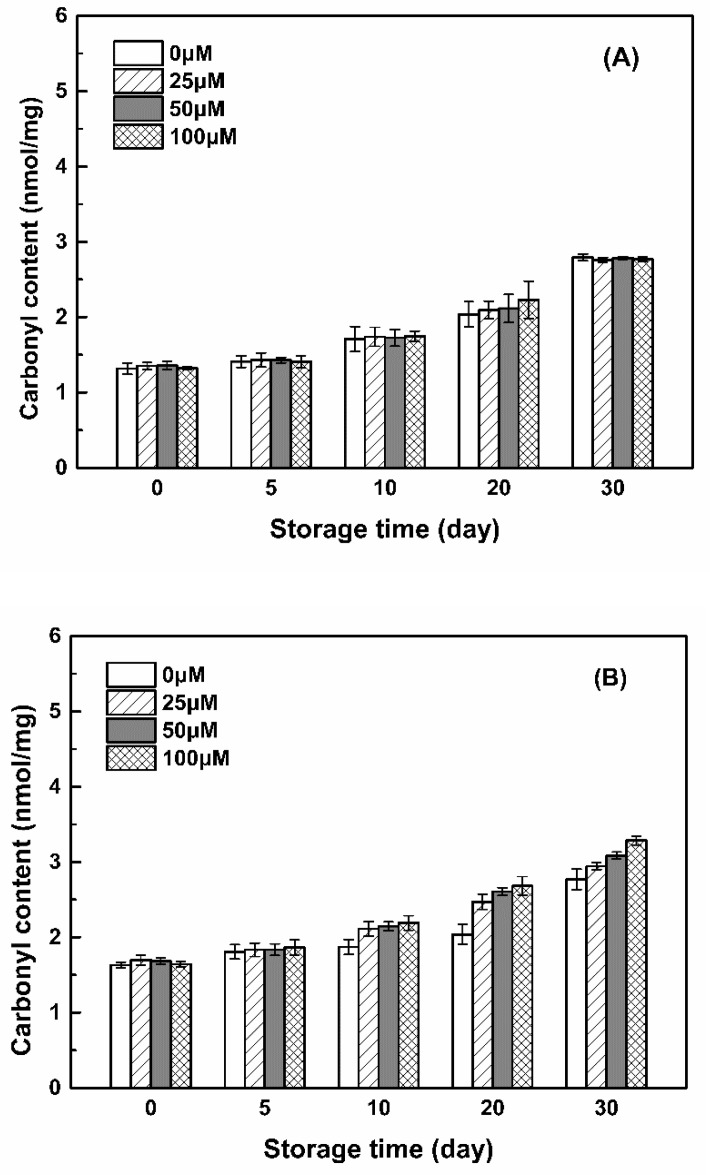

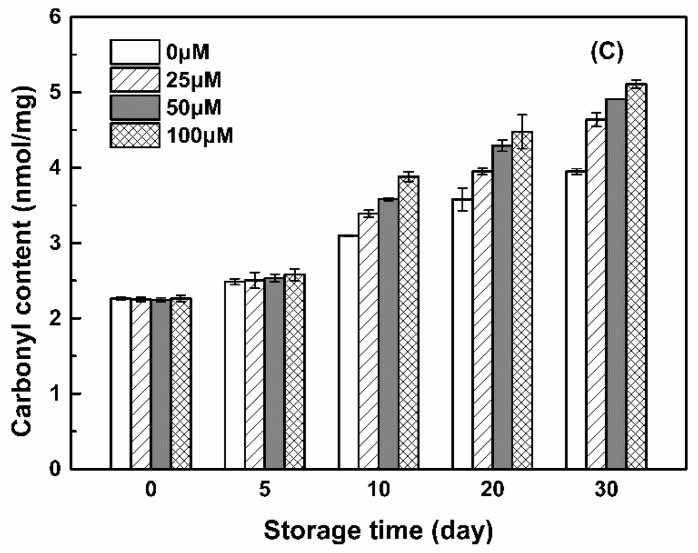
Carbonyl content of proteins in WPI-resveratrol (**A**), SC-resveratrol (**B**) and SPI-resveratrol (**C**) complex nanoparticles with various resveratrol concentrations during storage at 45 °C for 30 days. The concentration of proteins was 1%.

Carbonyl groups (aldehydes and ketones) are produced on the side chains of the protein when they are oxidized [49]. The carbonyl contents of WPI, SC and SPI were 1.32, 1.63 and 2.26 nmol/mg (Figure 8), respectively. Due to the preparation process of SC, it contains about 6% of small ions in addition to the pure casein, mainly calcium, phosphate, magnesium and citric acid [15], leading to the worst oxidation stability. The extraction of SPI from soy flour may accelerate its carbonylation [50], since soy protein is extremely vulnerable to the attack of peroxyl radicals, and its degree of oxidation is related to the residual lipid content and LOX activity during the preparation process [27,51]. The carbonyl content of WPI increased during storage, reaching 2.7 nmol/mg after 30 days, which was invariable with the addition of resveratrol (Figure 8). The carbonyl content of SPI and SC increased as the concentration of resveratrol increased after 10 days. When the resveratrol concentrations were 0, 25, 50 and 100 μM, the carbonyl content of SPI increased from 3.95 to 5.11 nmol/mg, while the carbonyl content of SC increased from 2.77 to 3.29 nmol/mg after 30 days. The increase in carbonyl content may be related to the formation of peroxides in the system, which is generated by oxygen molecules attacking free radicals. The formation of peroxides on the α-carbon or other carbons of protein amino acid residues will result in an increase in the carbonyl content [27]. From Figure 8, it indicated that the peroxide content in the three protein solutions was in the order of SPI > SC > WPI, and the addition of resveratrol to SC and SPI solutions produced more peroxides. Together with the sulfhydryl contents in Figure 7, these results indicated that the SPI was more labile to oxidation than SC in the presence of resveratrol.

#### 3.3.3. Amino Acid Composition

The oxidative attack of proteins modifies the side-chain groups of amino acid residues [52]. Table 1, Table 2 and Table 3 show the amino acid composition of WPI, SC and SPI in the absence and presence of resveratrol before and after storage for 30 days. The addition of resveratrol had no significant effect on the amino acid composition of the proteins before storage. The content of Cys ranked in order WPI > SPI > SC, and the surface and total sulfhydryl contents of SC were too low to be detected by the method of sulfhydryl analysis with DTNB (Figure 7). In the case of WPI alone, the content of Trp, Tyr, Thr, Lys, Met and Phe reduced after storage (Table 1), consistent with the indirect oxidation of WPI caused by the photodecomposition of folic acid [6]. Resveratrol had no effect on the change in the amino acid contents of WPI (Table 1). As for SC alone, the content of Trp, Tyr, Thr, Lys, Met, Asp and Arg reduced after storage and was more pronounced in the presence of resveratrol (Table 2). In addition, the content of Glu, Ser, Gly also reduced in the presence of resveratrol. The losses of Trp were about 11% for WPI and 79% and 87% for SC in the absence and presence of resveratrol, respectively. These results are consistent with a previous study that the tryptophan oxidation product, kynurenine, was higher in casein than β-LG upon photo-oxidation induced by riboflavin [4]. In the case of SPI alone, Asp, Ser, His, Gly, Thr, Tyr, Cys, Val, Met, Lys, Trp reduced after storage and was more pronounced in the presence of resveratrol (Table 3). In addition, the content of Glu also reduced in the presence of resveratrol. The reduction in the kinds and contents of total amino acids ranked in the order of SPI > SC > WPI (Table 1, Table 2 and Table 3).

### 3.4. Storage Stability of Resveratrol

By visual observation, all protein-resveratrol solutions were transparent and colorless except that SPI-resveratrol solutions were turbid at 1% protein (Table S1 and Figure S1). No significant change was observed for WPI-resveratrol solutions at 45 °C after 30 days. However, SPI-resveratrol and SC-resveratrol solutions changed from colorless to light yellow after storage. It has been reported that the wine with resveratrol changed from colorless to light yellow, due to its sensitivity to atmospheric oxidation [53]. After storage at 45 °C for 30 days, the total color difference (ΔE) and chroma change (ΔC*) of resveratrol alone increased, respectively, from 1.24 to 3.06 and from 1.17 to 2.94, as its concentration increased from 25 to 100 μM (Table 4). The ΔE and ΔC* of WPI, SC, SPI, and WPI-resveratrol solutions were less than those of resveratrol alone. However, the ΔE and ΔC* of SC-resveratrol and SPI-resveratrol solutions increased as the polyphenol concentration increased and were greater than the sum of correspondingly individual values at each concentration. A previous study also reported that WPI as emulsifier showed a better effect on inhibiting color changes of lutein-loaded emulsions relative to SC [54].

**Figure 9:**
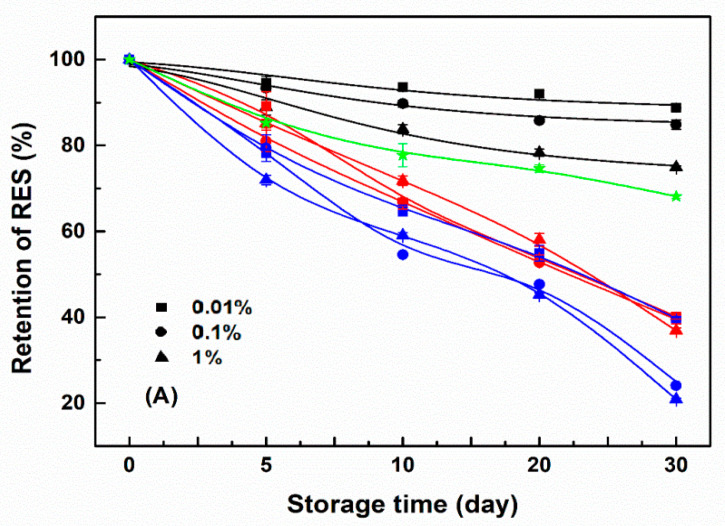

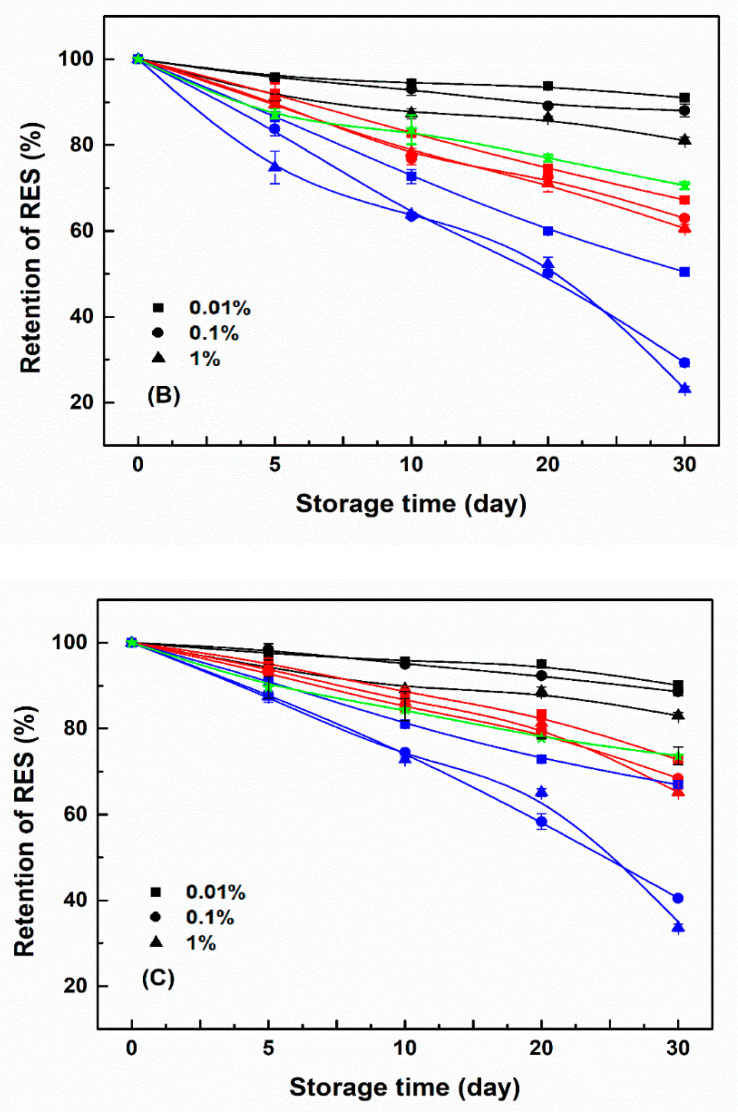
Retention of resveratrol alone (green) and in its WPI (black), SC (red) and SPI (blue) complex particles at various protein concentrations during storage at 45 °C. The concentrations of resveratrol were 25 (**A**), 50 (**B**) and 100 (**C**) μM.

Resveratrol alone degraded during storage at 45 °C and its content remained 68–74% after 30 days (Figure 9). The retention of resveratrol was improved by WPI, and the protective effect decreased slightly as the protein concentration increased. After 30 days of storage, the retention of resveratrol at 25 μM was around 88, 84, and 74% at 0.01, 0.1, and 1% WPI (Figure 9A), respectively, and the polyphenol retention was proportional to its initial concentration (Figure 9). In contrast, the loss of resveratrol was accelerated by SPI, the effect of which was more pronounced when the protein concentrations were 0.1% and 1% than 0.01% (Figure 9). SC also accelerated the degradation of resveratrol, the effect of which was less than that of SPI and decreased as the polyphenol concentration increased. The retention of resveratrol was consistent with the color change of its corresponding samples (Table 4).

## 4. Discussion

Resveratrol self-aggregates at a concentration higher than 40 μM, due to the hydrophobic stacking of aromatic phenol rings [55]. The aggregation of resveratrol reduces its contact with the external environment and affects its antioxidant activity with the highest value observed at a concentration of 30 μM [56]. Therefore, the retention of resveratrol increased from 68% to 74% as its concentration increased from 25 to 100 μM (Figure 9). α-Tocopherol (Log P ~ 8.84, https://go.drugbank.com/drugs/DB00163/ accessed on 8 July 2021), a hydrophobic vitamin E, was reported both bound in the molecular level and encapsulated as the aggregate in WPI particles, while naringenin (LogP ~ 2.84, https://go.drugbank.com/drugs/DB03467/ accessed on 8 July 2021), a polyhydroxy flavonoid, was bound in the molecular level [34]. Resveratrol (LogP ~ 3.4, https://go.drugbank.com/drugs/DB02709/ accessed on 8 July 2021) is more hydrophobic than naringenin but more hydrophilic than α-tocopherol. When calculated, based on the loading efficiency of resveratrol in Figure 5, the encapsulated amount of resveratrol in protein particles increased as the polyphenol concentration increased (Figure S3). It is thus possible that the aggregated resveratrol in protein particles increased as its concentration increased, which was supported by the transfer from the additive to the masking effect of total antioxidant activity (Figure 6). Therefore, the polyphenol retention increased with its concentration in protein particles (Figure 9).

For WPI, the solvent-accessible (bounded in the molecular level and in free state) resveratrol can scavenge and control the free radicals in the system within a certain range. Its oxidation was the least and not affected by resveratrol during storage for 30 days (Figure 7 and Figure 8 and Table 1). At the same time, the stability of resveratrol was improved by WPI, with a retention of above 74% after 30 days (Figure 9). It is thus speculated that there is no reciprocal oxidation between WPI and resveratrol during storage. As the concentration of WPI increased, the loading efficiency of resveratrol increased (Figure 5), but the polyphenol stability decreased (Figure 9). These results suggest that the loaded microenvironment is not conducive to the polyphenol stability, compared to the free part in the WPI solution. The protective effect of WPI on resveratrol stability might not be attributed to the complex property of the protein.

For SPI, the encapsulated resveratrol located in the hydrophobic core could not exert its antioxidant capacity. Thus its oxidation was the most at the beginning and accelerated by resveratrol during storage after 10 days (Figure 7 and Figure 8 and Table 3). At the same time, the stability of resveratrol decreased upon loading in SPI particles (Figure 9). These results suggest the occurrence of reciprocal oxidation between SPI and resveratrol. The co-oxidation has been reported for whey protein and Antarctic krill oil in oil-in-water emulsion [57]. The initial state of the SPI system contained more peroxides than SC and WPI (Figure 7 and Figure 8), free radicals and hydroperoxides generated during protein oxidation may accelerate the degradation of resveratrol [58] (Figure 9). It has also been reported that ascorbic acid acted as a co-oxidant by generating superoxide anions in the presence of air and extracting hydrogen from the carrier [59]. Resveratrol is oxidized to generate H_2_O_2_ [60]. When the retention of resveratrol was between 59 and 73% after 10 days (Figure 9), the polyphenol may act as a co-oxidant to accelerate the oxidation of SPI (Figure 8).

However, most of the resveratrol in the SC system was encapsulated in the hydrophobic core of the protein, but also partially bounded with submicelles in the molecular level, which can play their antioxidant effect to a certain extent. The oxidation of SC was more pronounced than that of WPI but less than that of SPI at the beginning and during storage in the absence and presence of resveratrol (Figure 7 and Figure 8 and Table 2). At the same time, the impact of SC on resveratrol stability basically changed from a protective to a harmful effect during storage (Figure 9). The antioxidant activity of SC was greater than that of WPI and SPI (Figure 6), and the loading efficiencies of resveratrol in SC particles were greater than those in SPI and WPI particles at protein concentrations of 0.1% and 1% (Figure 5). Therefore, the stability of resveratrol was initially improved by SC (Figure 9). A stable protein carrier can maintain the stability of polyphenols through scavenging free radicals and isolating the interference of external unfavorable factors [61]. Then, with the increasing oxidation of SC, the ability to scavenge free radicals was not enough to resist the auto-oxidation of SC. The system was out of balance and the protein changed from antioxidant to pro-oxidant to cause the co-oxidation with resveratrol (Figure 8 and Figure 9).

According to the molecular mechanism of the protein–polyphenol interaction, the di-phenol part of polyphenol is easily oxidized by molecular oxygen and side-chain amino groups under certain conditions to form quinine, which can form a dimer in a side reaction and interact with the amino group of polypeptide or the irreversible reaction of the sulfhydryl side chain leads to the formation of protein cross-links. The closer the distance between the formed oxidation product and the α-carbon or other carbons of protein amino acid residues, the more easily the reaction occurs (Figure 4). Meanwhile, quinine can undergo condensation reactions to form high molecular weight, highly reactive brown tannins [17], which is verified in Table 4 and Figure S1. The formation of a covalent EGCG-protein complex involved the reaction of dimer quinone with protein nucleophilic side chains, such as lysine and cysteine residues, which is consistent with the results of amino acid composition in SC/SPI-resveratrol complex particles after storage (Table 1, Table 2 and Table 3). It has been assumed that the structure of SC and SPI gradually became flexible during storage and the exposed active groups benefited from the covalent interactions of protein-resveratrol complexations [62].

## 5. Conclusions

WPI improved the storage stability of resveratrol, but SPI accelerated the loss of resveratrol, while the impact of SC on resveratrol stability basically changed from a protective to a harmful effect. The stability of polyphenols increased as the polyphenol concentration increased but decreased as the protein concentration increased. The loading efficiency of resveratrol in protein particles and the initial antioxidant activity of proteins were not the dominant factors to affect the storage stability of resveratrol. The effect of proteins on the stability of resveratrol was mainly dependent on their oxidation sensitivity. The co-oxidation of resveratrol with SPI and SC occurred during storage. The oxidation degree of WPI was the least and not affected by resveratrol. The results obtained suggest that WPI might be a better material to design an effective carrier for the long-term protection of resveratrol than SPI and SC. To our knowledge, it is the first time that the important role of protein oxidability on the stability of polyphenols during storage has been reported and provides useful guidelines for the long-term protection of polyphenols by protein-based carriers.

## Figures and Tables

**Table 1:** Amino acid composition of whey protein isolate (WPI) at 1% in the absence and presence of 100 μM resveratrol (RES) before and after storage for 30 days.

Amino Acid	Content of Amino Acid (μg/mL)			
	WPI (0)	WPI-RES (0)	WPI (30)	WPI-RES (30)
Cys	121 ± 4 ^a^	119 ± 4 ^a^	106 ± 4 ^b^	104 ± 3 ^b^
Trp	198 ± 7 ^a^	195 ± 9 ^a^	172 ± 8 ^b^	179 ± 6 ^b^
Tyr	298 ± 9 ^a^	301 ± 7 ^a^	277 ± 6 ^b^	280 ± 6 ^b^
Thr	433 ± 5 ^a^	430 ± 7 ^a^	417 ± 8 ^b^	410 ± 8 ^b^
Lys	942 ± 17 ^a^	947 ± 12 ^a^	933 ± 11 ^b^	928 ± 9 ^b^
Met	194 ± 5 ^a^	192 ± 7 ^a^	165 ± 5 ^b^	163 ± 8 ^b^
Phe	346 ± 2 ^a^	351 ± 6 ^a^	324 ± 8 ^b^	320 ± 5 ^b^
Asp	1195 ± 16 ^a^	1203 ± 11 ^a^	1196 ± 38 ^a^	1158 ± 43 ^a^
Arg	233 ± 11 ^a^	235 ± 8 ^a^	239 ± 8 ^a^	235 ± 9 ^a^
Glu	1790 ± 17 ^a^	1784 ± 29 ^a^	1777 ± 17 ^a^	1783 ± 26 ^a^
Ser	296 ± 7 ^a^	292 ± 5 ^a^	295 ± 8 ^a^	299 ± 9 ^a^
Gly	161 ± 2 ^a^	158 ± 4 ^a^	160 ± 3 ^a^	158 ± 3 ^a^
His	172 ± 2 ^a^	169 ± 3 ^a^	172 ± 5 ^a^	173 ± 6 ^a^
Val	506 ± 14 ^a^	496 ± 29 ^a^	508 ± 13 ^a^	495 ± 10 ^a^
Ala	474 ± 7 ^a^	474 ± 5 ^a^	475 ± 5 ^a^	472 ± 9 ^a^
Ile	586 ± 18 ^a^	579 ± 16 ^a^	573 ± 10 ^a^	575 ± 15 ^a^
Leu	991 ± 16 ^a^	981 ± 22 ^a^	980 ± 12 ^a^	976 ± 20 ^a^
Pro	399 ± 19 ^a^	393 ± 7 ^a^	398 ± 28 ^a^	406 ± 10 ^a^
Total	9335 ± 74 ^a^	9299 ± 53 ^a^	9167 ± 43 ^b^	9114 ± 64 ^b^

Note: Different lower-case letters in the same row represent significantly different mean values (*p* < 0.05).

**Table 2:** Amino acid composition of sodium caseinate (SC) in the absence and presence of resveratrol (RES) before and after storage for 30 days.

Amino Acid	Content of Amino Acid (μg/mL)			
	SC (0)	SC-RES (0)	SC (30)	SC-RES (30)
Cys	5 ± 0 ^a^	5 ± 0 ^a^	5 ± 0 ^a^	4 ± 1 ^a^
Trp	571 ± 9 ^a^	566 ± 12 ^a^	119 ± 14 ^b^	74 ± 10 ^c^
Tyr	451 ± 9 ^a^	448 ± 12 ^a^	409 ± 10 ^b^	384 ± 6 ^c^
Thr	344 ± 5 ^a^	350 ± 8 ^a^	330 ± 3 ^b^	318 ± 2 ^c^
Lys	744 ± 7 ^a^	737 ± 11 ^a^	690 ± 9 ^b^	629 ± 7 ^c^
Met	226 ± 8 ^a^	222 ± 2 ^a^	185 ± 2 ^b^	184 ± 2 ^b^
Phe	443 ± 8 ^a^	439 ± 6 ^a^	427 ± 10 ^a^	433 ± 10 ^a^
Asp	548 ± 14 ^a^	556 ± 12 ^a^	493 ± 18 ^b^	460 ± 13 ^c^
Arg	337 ± 10 ^a^	345 ± 9 ^a^	313 ± 12 ^b^	294 ± 3 ^c^
Glu	2053 ± 87 ^a^	2014 ± 79 ^a^	2048 ± 57 ^a^	1998 ± 50 ^b^
Ser	380 ± 7 ^a^	378 ± 5 ^a^	384 ± 8 ^a^	375 ± 8 ^b^
Gly	159 ± 4 ^a^	153 ± 9 ^a^	160 ± 2 ^a^	152 ± 3 ^b^
His	293 ± 3 ^a^	290 ± 9 ^a^	299 ± 7 ^a^	288 ± 7 ^a^
Val	600 ± 10 ^a^	593 ± 19 ^a^	588 ± 15 ^a^	589 ± 11 ^a^
Ala	260 ± 7 ^a^	263 ± 7 ^a^	252 ± 4 ^a^	252 ± 4 ^a^
Ile	487 ± 12 ^a^	479 ± 12 ^a^	473 ± 10 ^a^	467 ± 16 ^a^
Leu	788 ± 14 ^a^	795 ± 22 ^a^	783 ± 20 ^a^	780 ± 17 ^a^
Pro	727 ± 16 ^a^	722 ± 10 ^a^	720 ± 18 ^a^	735 ± 11 ^a^
Total	9416 ± 106 ^a^	9355 ± 99 ^a^	8678 ± 70 ^b^	8416 ± 68 ^c^

Note: Different lower-case letters in the same row represent significantly different mean values (*p* < 0.05).

**Table 3:** Amino acid composition of soy protein isolate (SPI) in the absence and presence of resveratrol (RES) before and after storage for 30 days.

Amino Acid	Content of Amino Acid (μg/mL)			
	SPI (0)	SPI-RES (0)	SPI (30)	SPI-RES (30)
Cys	20 ± 2 ^a^	19 ± 4 ^a^	13 ± 2 ^b^	5 ± 2 ^c^
Trp	160 ± 6 ^a^	158 ± 8 ^a^	93 ± 4 ^b^	78 ± 3 ^c^
Tyr	281 ± 8 ^a^	276 ± 6 ^a^	231 ± 4 ^b^	200 ± 1 ^c^
Thr	209 ± 3 ^a^	211 ± 5 ^a^	177 ± 8 ^b^	170 ± 3 ^c^
Lys	481 ± 7 ^a^	476 ± 8 ^a^	403 ± 10 ^b^	382 ± 8 ^c^
Met	84 ± 3 ^a^	89 ± 8 ^a^	79 ± 4 ^b^	63 ± 6 ^c^
Phe	418 ± 6 ^a^	421 ± 9 ^a^	358 ± 10 ^b^	347 ± 7 ^c^
Asp	735 ± 14 ^a^	735 ± 13 ^a^	685 ± 21 ^b^	554 ± 10 ^c^
Arg	585 ± 5 ^a^	591 ± 6 ^a^	584 ± 5 ^a^	591 ± 8 ^a^
Glu	1520 ± 67 ^a^	1479 ± 67 ^a^	1335 ± 79 ^a^	1192 ± 106 ^b^
Ser	371 ± 9 ^a^	365 ± 8 ^a^	325 ± 9 ^b^	300 ± 4 ^c^
Gly	352 ± 8 ^a^	346 ± 9 ^a^	328 ± 8 ^b^	280 ± 9 ^c^
His	213 ± 6 ^a^	215 ± 3 ^a^	195 ± 6 ^b^	167 ± 3 ^c^
Val	431 ± 8 ^a^	442 ± 13 ^a^	399 ± 6 ^b^	352 ± 7 ^c^
Ala	356 ± 4 ^a^	350 ± 9 ^a^	355 ± 11 ^a^	349 ± 6 ^a^
Ile	416 ± 11 ^a^	409 ± 7 ^a^	414 ± 3 ^a^	402 ± 8 ^a^
Leu	623 ± 12 ^a^	627 ± 7 ^a^	614 ± 10 ^a^	606 ± 19 ^a^
Pro	366 ± 6 ^a^	357 ± 11 ^a^	358 ± 8 ^a^	347 ± 10 ^a^
Total	7621 ± 90 ^a^	7566 ± 89 ^a^	6946 ± 98 ^b^	6385 ± 121 ^c^

Note: Different lower-case letters in the same row represent significantly different mean values (*p* < 0.05).

**Table 4:** Total color difference (ΔE) and chroma change (ΔC*) of WPI-resveratrol, SC-resveratrol and SPI-resveratrol complex solutions before and after storage at 45 °C for 30 days. The concentration of proteins was 1%.

Protein	Concentration of Resveratrol (μM)			
	0	25	50	100
ΔE				
		1.24 ± 0.19 ^Aa^	1.85 ± 0.45 ^Aa^	3.06 ± 0.27 ^Bb^
WPI	0.74 ± 0.65 ^Aa^	0.59 ± 0.31 ^Aa^	0.58 ± 0.47 ^Aa^	0.87 ± 0.92 ^Aa^
SPI	1.16 ± 0.70 ^Aa^	3.79 ± 0.83 ^Bb^	6.95 ± 0.96 ^Cb^	9.16 ± 1.54 ^Dc^
SC	0.30 ± 0.20 ^Aa^	2.82 ± 1.19 ^Bb^	6.04 ± 1.49 ^Cb^	8.59 ± 0.71 ^Dc^
ΔC*				
		1.17 ± 0.15 ^Aa^	1.73 ± 0.35 ^Ba^	2.94 ± 0.23 ^Cb^
WPI	0.29 ± 0.22 ^Aa^	0.56 ± 0.31 ^Aa^	0.42 ± 0.25 ^Aa^	0.30 ± 0.12 ^Aa^
SPI	0.58 ± 0.47 ^Aa^	3.30 ± 0.46 ^Bb^	5.94 ± 0.43 ^Cb^	8.31 ± 1.39 ^Dd^
SC	0.14 ± 0.09 ^Aa^	2.63 ± 1.02 ^Bb^	4.44 ± 1.23 ^Cb^	6.19 ± 0.50 ^Dc^

Note: Different lower-case letters in the same column represent significantly different mean values, different upper-case letters in the same row represent significant different mean values (*p* < 0.05).

## Data Availability

The data are contained within the article and supplementary materials.

## Supplementary Materials

The following supporting information can be downloaded at: https://www.mdpi.com/article/10.3390/antiox11040647/s1, Figure S1: Appearance of WPI-resveratrol, SC-resveratrol and SPI-resveratrol complex nanoparticles before (A–C, respectively) and after (a–c, respectively) storage at 45 °C for 30 days. The concentration of proteins from left to right was 0.01%, 0.1% and 1%. The concentrations of resveratrol from left to right was 25, 50 and 100 μM; Figure S2: XRD patterns of resveratrol (black), proteins (blue), their physical mixtures (red) and resveratrol-loaded protein particles (green). The concentration of protein was 1%; Figure S3: Loading efficiency of resveratrol in its complex particles with WPI (black), SC (red) and SPI (blue) at 0.01%, 0.1% and 1% after storage at 45 °C; Table S1: Turbidity of WPI-resveratrol, SC-resveratrol and SPI-resveratrol complex nanoparticles at various concentrations of proteins and resveratrol.

## Abbreviations

WPI	whey protein isolate
SC	sodium caseinate
SPI	soy protein isolate
BSA	bovine serum albumin
RES	resveratrol
GRAS	generally recognized as safe
ABTS	2,2′-azino-bis-3- ethylbenzthiazoline-6-sulphonic acid
CMC	critical micelle concentration
β-LG	β-lactoglobulin
LOX	lipoxygenase
SH	sulfhydryl
ΔE	total color difference
ΔC*	chroma change
