# Supplementary material for: Resveratrol Stabilization and Loss by Sodium Caseinate, Whey and Soy Protein Isolates: Loading, Antioxidant Activity, Oxidability
Source: Antioxidants (Basel). 2022 Mar 28;11(4):647. doi: 10.3390/antiox11040647 (PMC9030250; doi:10.3390/antiox11040647)
Supplement: Supplementary file 1 [file antioxidants-11-00647-s001.zip › antioxidants-1636191-SI.pdf]

Supplementary Data

Mechanism of resveratrol stabilization and loss by sodium ca-seinate, whey and soy protein isolates: loading, antioxidant ac-tivity, oxidability

Xin Yin[1,2], Hao Cheng[1,2], Wusigale[3,4], Huanhuan Dong[1,2], Li Liang[1,2*]

[1]State Key Lab of Food Science and Technology, Jiangnan University, Wuxi, Jiangsu, China

[2]School of Food Science and Technology, Jiangnan University, Wuxi, Jiangsu, China

[3]Key Laboratory of Dairy Biotechnology and Engineering, Ministry of Education, Inner Mongolia Agricultural University, Hohhot, Inner Mongolia, China

[4]Inner Mongolia Key Laboratory of Dairy Biotechnology and Engineering, Hohhot, Inner Mongolia, China

[*]Corresponding author: Li Liang

Address: State Key Lab of Food Science and Technology, School of Food Science and Technology, Jiangnan University, Wuxi, Jiangsu 214122, China.

Telephone: +86(510)8519-7367;

Email: liliang@jiangnan.edu.cn.

Turbidity was determined from the transmission at 500 nm according the method by (Liang et al., 2011), using a UV-1800 UV-Vis spectrophotometer (Shimadzu Co., Tokyo, Japan) and expressed as (100-% transmission).

Table S1. Turbidity of WPI-resveratrol, SC-resveratrol and SPI-resveratrol complex nanoparticles at various concentrations of proteins and resveratrol.

Concentration of proteins (%)	Concentration of resveratrol (μM)			
	0	25	50	100
		WPI		
0.01	0.29±0.11 ^{Aa}	0.79±0.11 ^{Ba}	0.60±0.10 ^{Ba}	0.81±0.14 ^{Ba}
0.1	0.51±0.07 ^{Ab}	0.90±0.15 ^{Ba}	0.97±0.02 ^{Bb}	0.82±0.21 ^{Ba}
1	4.96±0.13 ^{Ac}	5.00±0.34 ^{Ab}	4.82±0.27 ^{Ac}	4.74±0.18 ^{Ab}
		SC		
0.01	0.27±0.03 ^{Aa}	0.35±0.03 ^{Ba}	0.35±0.08 ^{Ba}	0.33±0.01 ^{Ba}
0.1	2.19±0.14 ^{Ab}	2.05±0.18 ^{Ab}	2.00±0.14 ^{Ab}	2.01±0.09 ^{Ab}
1	17.82±0.13 ^{Ac}	17.86±0.30 ^{Ac}	17.80±0.07 ^{Ac}	17.58±0.07 ^{Ac}
		SPI		
0.01	0.98±0.09 ^{Aa}	1.92±0.48 ^{Ba}	1.88±0.49 ^{Ba}	2.07±0.28 ^{Ba}
0.1	9.19±0.08 ^{Ab}	13.70±1.72 ^{Bb}	13.78±1.81 ^{Bb}	13.90±1.83 ^{Bb}
1	51.50±0.19 ^{Ac}	51.60±2.41 ^{Ac}	51.66±2.12 ^{Ac}	51.04±2.12 ^{Ac}

Note: Different lower case letters in the same column represent different significance levels, different upper case letters in the same row represent different significance levels ($P < 0.05$).

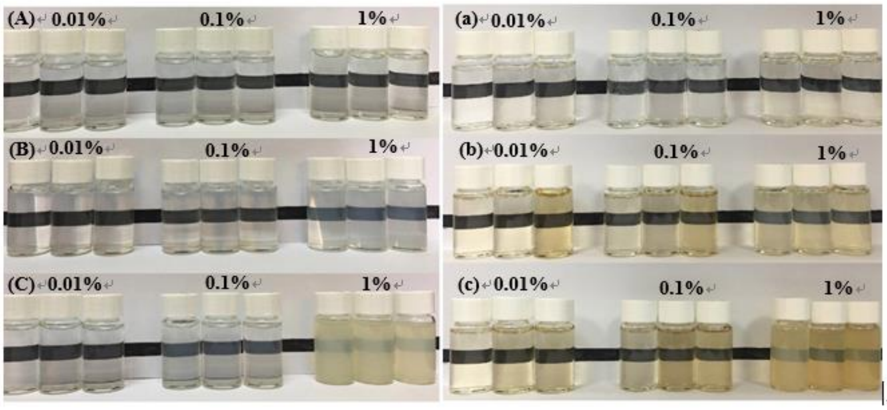

Figure S1. Appearance of WPI-resveratrol, SC-resveratrol and SPI-resveratrol complex nanoparticles before (A-C, respectively) and after (a-c, respectively) storage at 45°C for 30 days. The concentration of proteins from left to right were 0.01%, 0.1% and 1%. The concentrations of resveratrol from left to right were 25, 50 and 100 μM.

X-ray diffraction

X-ray diffractogram of resveratrol, proteins, resveratrol-loaded protein particles, physical mixture of protein and resveratrol were analyzed by using a Bruker D2 PHASER X-ray diffractometer (Brucker, Odelzhausen, Germany) equipped with a copper anode that produces Cu Kα radiation. The 2θ angle was set from 3° to 60° in continuous mode using a step size of 0.02° and step time of 5 s and measured at 40 kV of an accelerating voltage and 40 mA of a tube current.

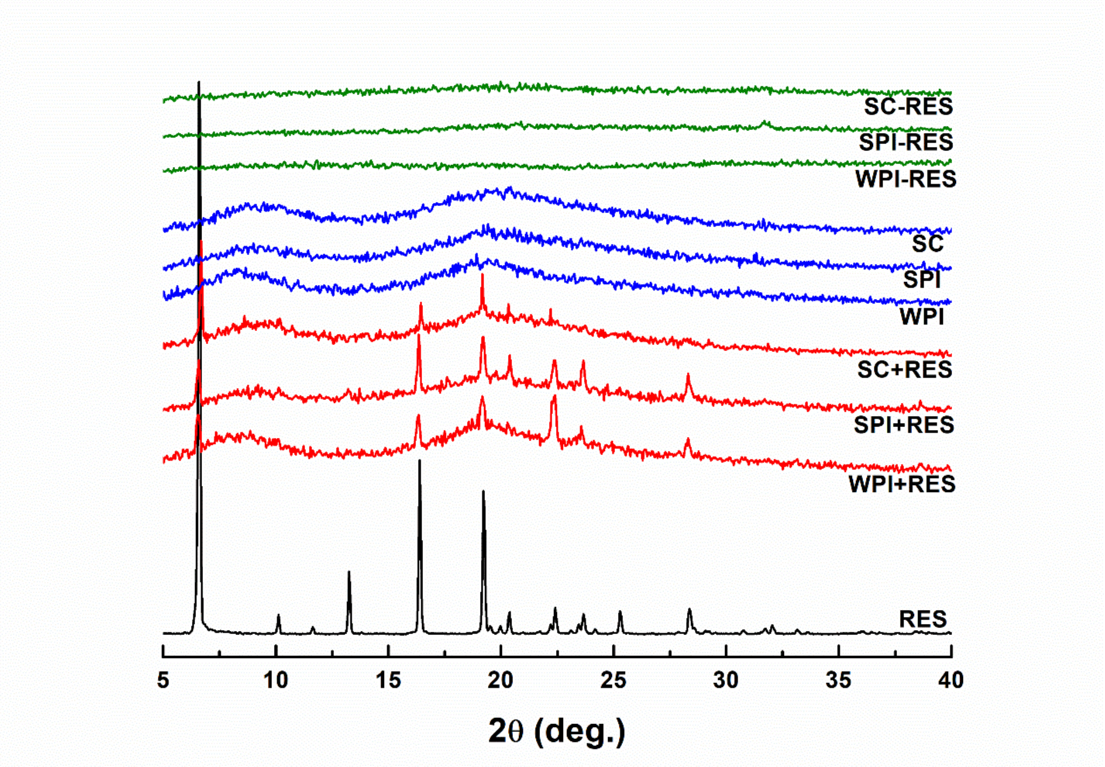

Figure S2. XRD patterns of resveratrol (black), proteins (blue), their physical mixtures (red) and resveratrol-loaded protein particles (green). The concentration of protein was 1%.

Figure S3

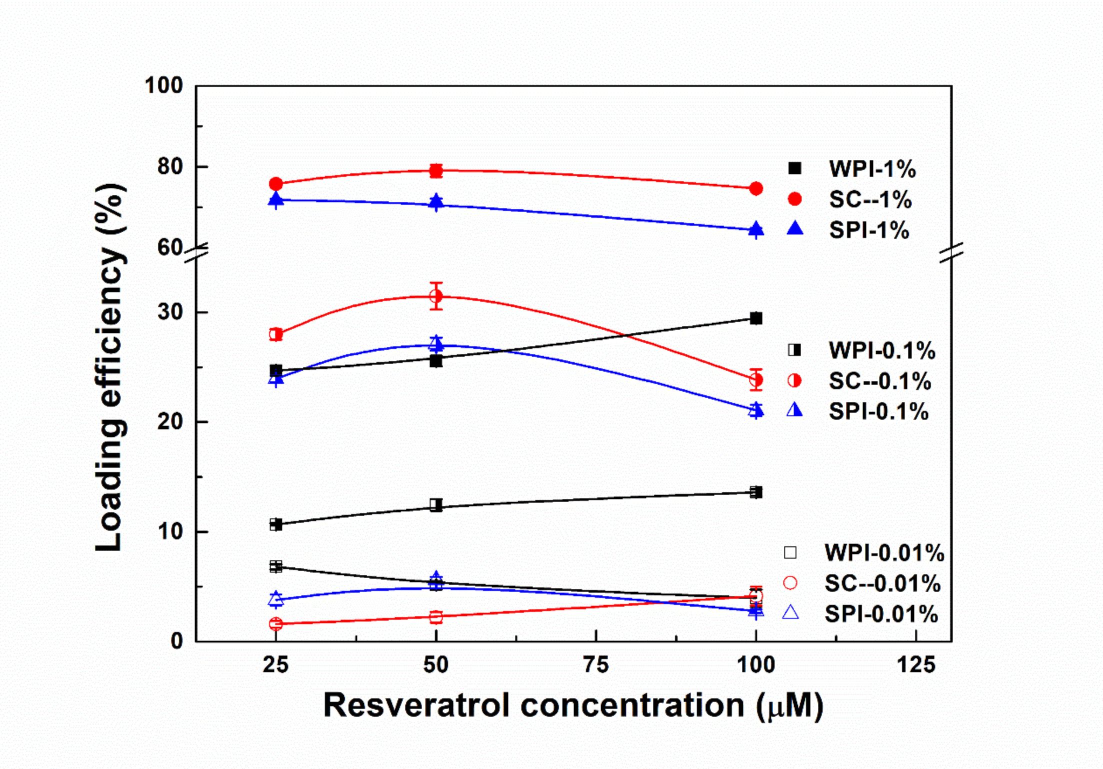

Figure S3. Loading efficiency of resveratrol in its complex particles with WPI (black), SC (red) and SPI (blue) at 0.01%, 0.1% and 1% after storage at 45°C.
